# Supplementary material for: Migration and Interaction in a Contact Zone: mtDNA Variation among Bantu-Speakers in Southern Africa
Source: PLoS One. 2014 Jun 5;9(6):e99117. doi: 10.1371/journal.pone.0099117 (PMC4047067; doi:10.1371/journal.pone.0099117)

Supporting Information Figure S1: Schematic phylogeny of the African haplogroups found in the 982 individuals of our dataset. The reference sequences RSRS (Behar et al. 2012) and rCRS (corresponding to European haplogroup H, Andrews et al. 1999) are indicated with a black line. Dashed lines indicate nodes with low posterior probability for which the topology could not be resolved. The width of the triangles is proportional to the frequency of a given haplogroup in the dataset. Haplogroups L3d and L3f, in yellow, are described in more detail in Figure S4A and S4B.

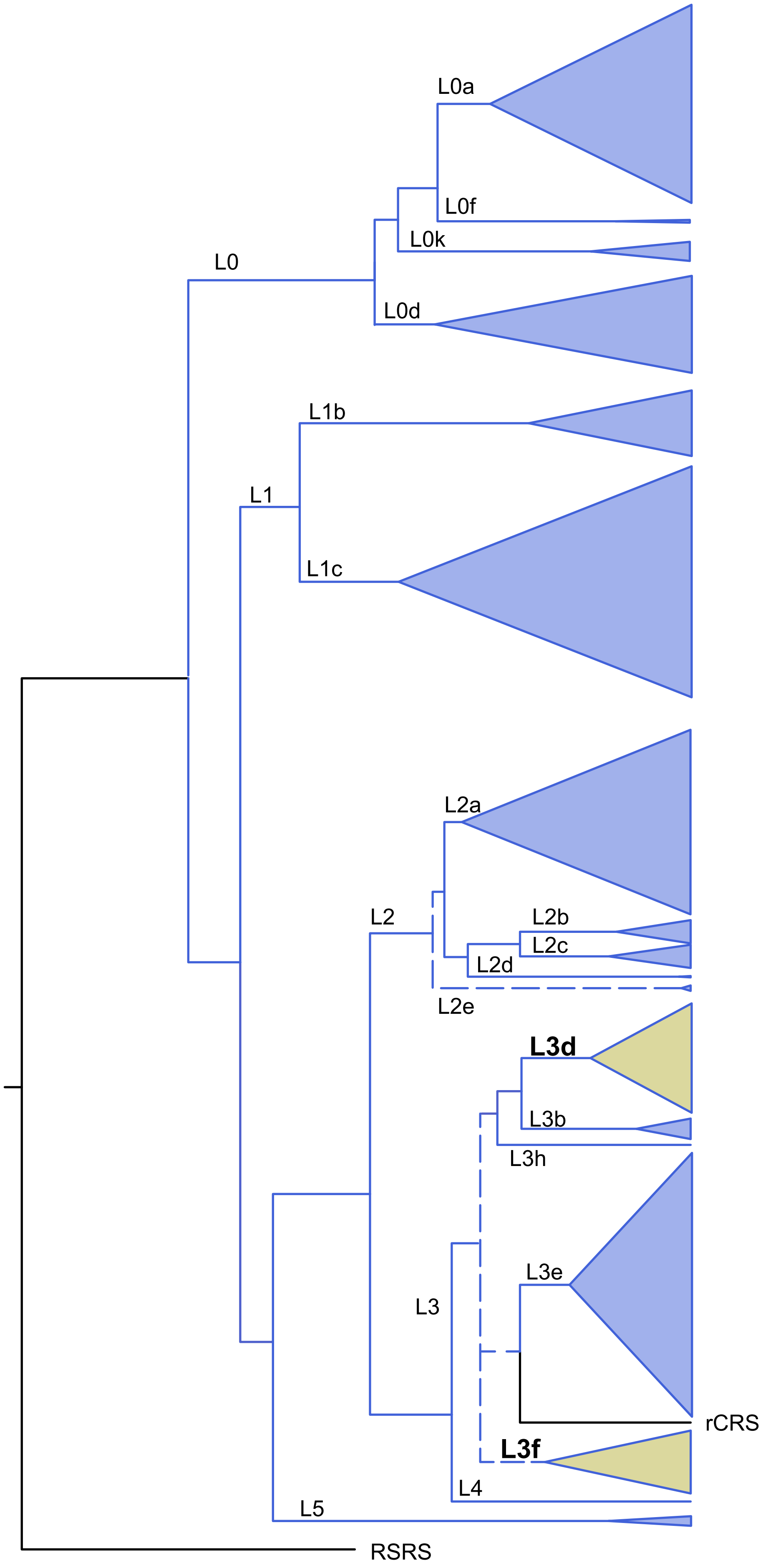

Supporting InformationFigure S2: CA plots based on basic haplogroup frequencies. Color coding by linguistic affiliation as specified in Table 1. A: Including all haplogroups and all populations. B: Including all haplogroups but excluding outlier populations. C: Excluding haplogroups L0d and L0k and excluding outliers.

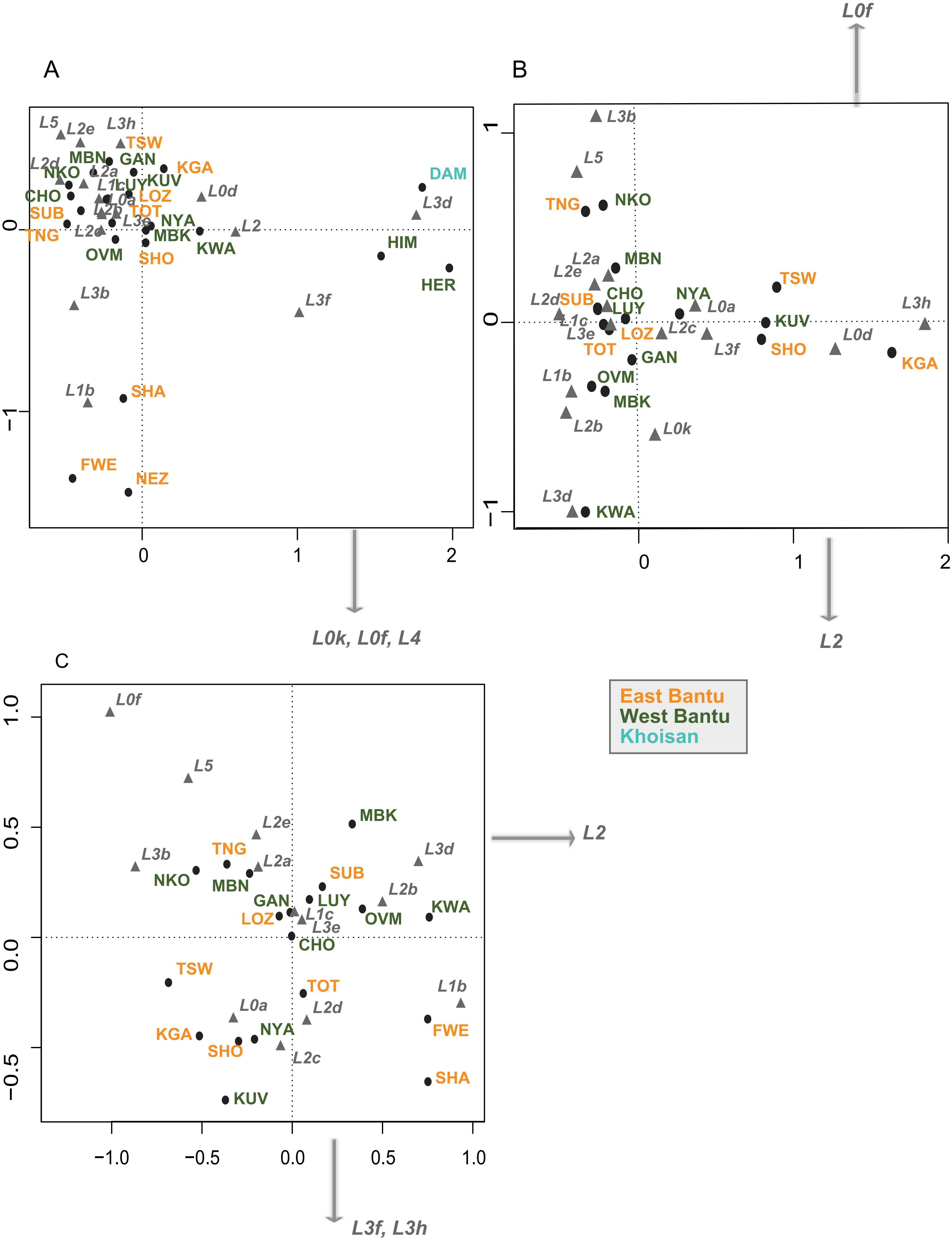

Supporting Information Figure S3: Haplogroup frequencies of important haplogroups in the populations studied here. A: Haplogroups L0d and L0k. B. Subhaplogroups L3d3a1 and L3f1b4a. The size of the circles is proportional to the sample size.

A

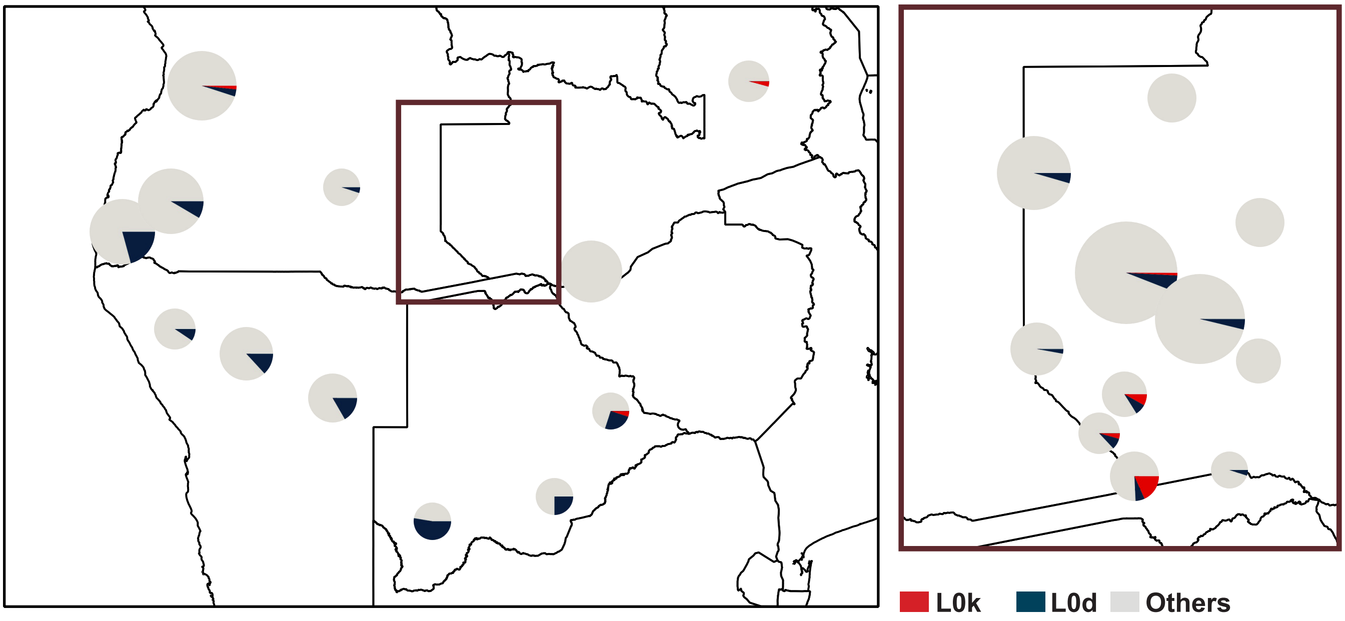

B

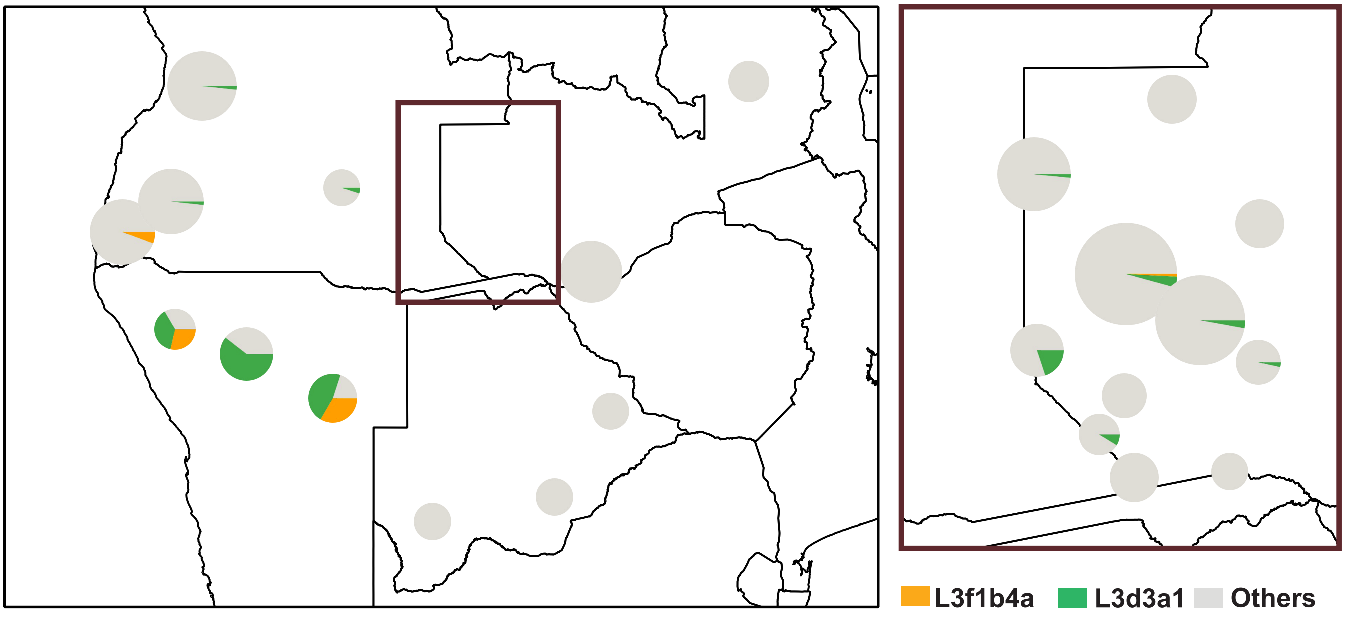

Supporting Information Figure S4A: Bayesian tree of all African complete mtDNA sequences belonging to haplogroup L3d, and S4B: belonging to haplogroup L3f. The sequences included in these trees are the same as those included in the networks in Figure 4B and D. Branches discussed in the main text are highlighted. The posterior probability associated with major nodes is indicated in red font. Note the different time scale between the two trees.

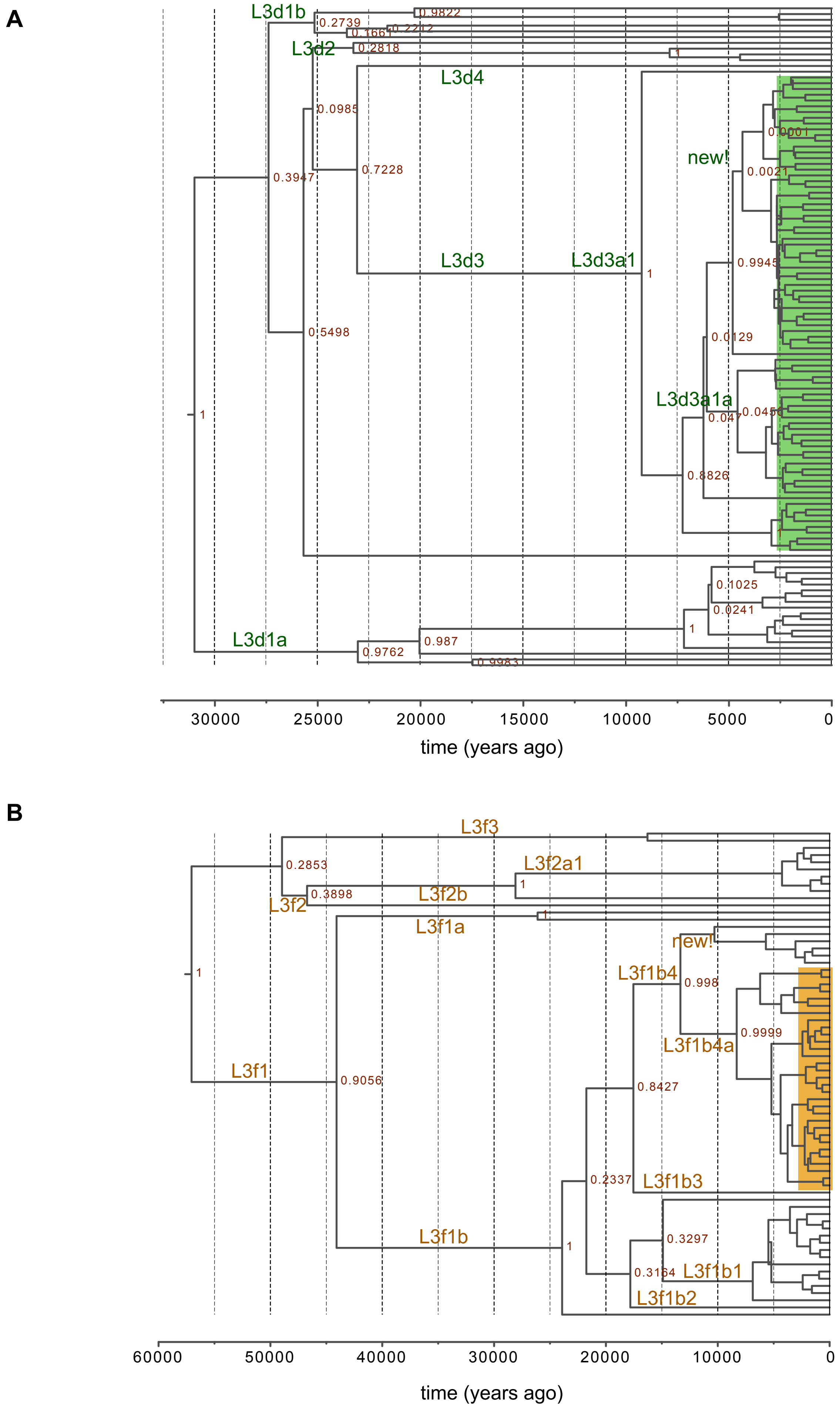

Supporting Information Figure S5: Bayesian Skyline Plots for the Himba, Herero, Kuvale and Damara. X axis: time in years ago. Y axis: effective population size per generation time.

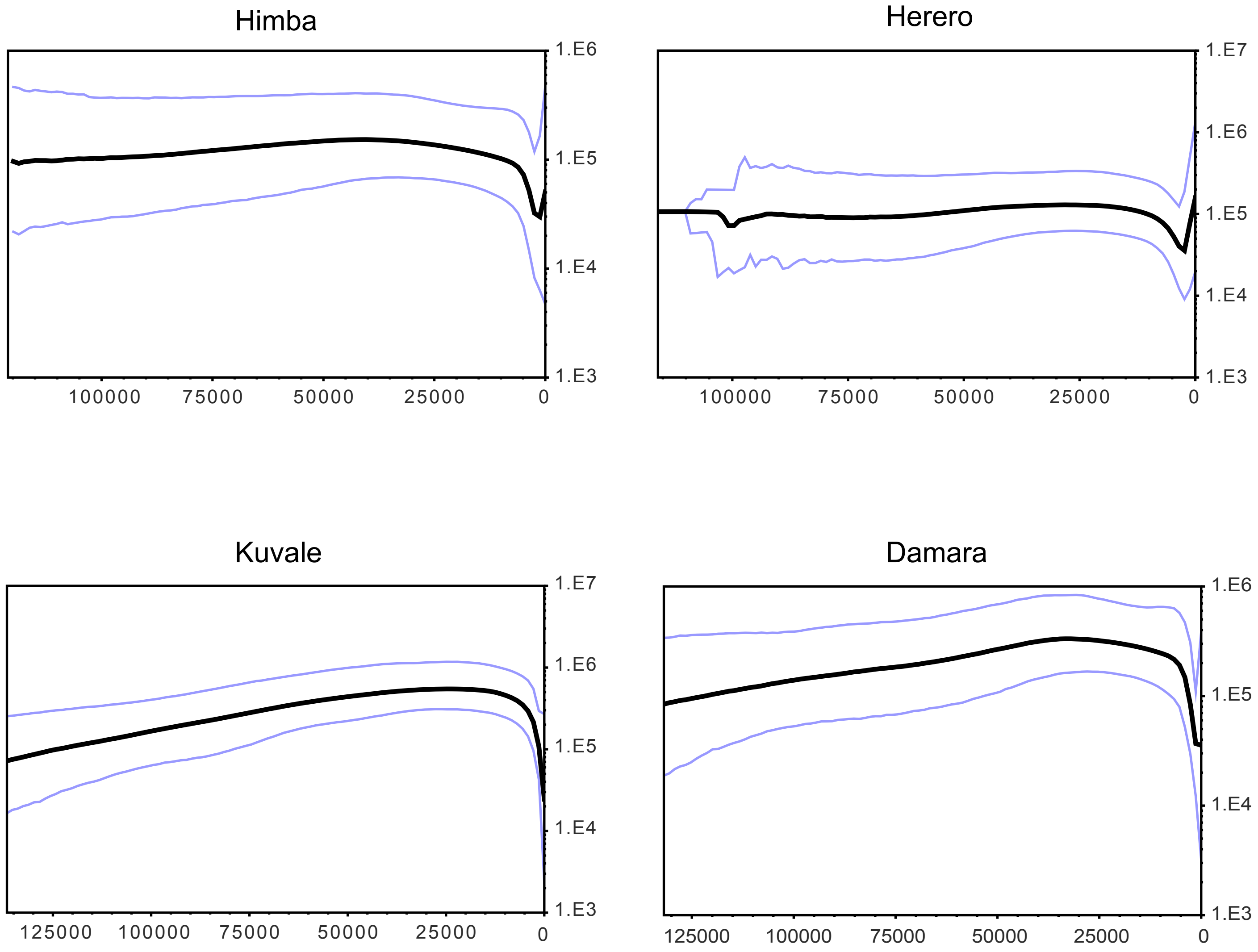

Supplement: File S1 — Contains the files: Figure S1: Schematic phylogeny of the African haplogroups found in the 982 individuals of our dataset. The reference sequences RSRS (Behar et al. 2012) and rCRS (corresponding to European haplogroup H, Andrews et al. 1999) are indicated with a black line. Dashed lines indicate nodes with low posterior probability for which the topology could not be resolved. The width of the triangles is proportional to the frequency of a given haplogroup in the dataset. Haplogroups L3d and L3f, in yellow, are described in more detail in Figure S4A and S4B. Figure S2: CA plots based on basic haplogroup frequencies. Color coding by linguistic affiliation as specified in Table 1. A: Including all haplogroups and all populations. B: Including all haplogroups but excluding outlier populations. C: Excluding haplogroups L0d and L0k and excluding outliers. Figure S3: Haplogroup frequencies of important haplogroups in the populations studied here. A: Haplogroups L0d and L0k. B. Subhaplogroups L3d3a1 and L3f1b4a. The size of the circles is proportional to the sample size. Figure S4A: Bayesian tree of all African complete mtDNA sequences belonging to haplogroup L3d, and S4B: belonging to haplogroup L3f. The sequences included in these trees are the same as those included in the networks in Figure 4B and D. Branches discussed in the main text are highlighted. The posterior probability associated with major nodes is indicated in red font. Note the different time scale between the two trees. Figure S5: Bayesian Skyline Plots for the Himba, Herero, Kuvale and Damara. X axis: time in years ago. Y axis: effective population size per generation time. (PDF) [file pone.0099117.s001.pdf]
